# Diagnosis and Molecular Characterization of Dermatophytosis: An Observational Study

**DOI:** 10.12688/f1000research.141657.2

**Published:** 2024-07-08

**Authors:** Aditi Warghade, Gargi Mudey

**Affiliations:** 1 Department of Microbiology, Jawaharlal Nehru Medical College, Datta Meghe Institute of Higher Education and Research, Wardha, Maharashtra, 442001, India

**Keywords:** Dermatophytosis, Molecular, Characterisation, RAPD-PCR, Electrophoresis, fungus, keratinophilic, Conventional

## Abstract

Dermatophytes are the keratinophilic fungi which infect humans and is the most recurring type of disease. The high level of transmissibility creates an epidemiological risk and emphasises the significance of these illnesses. However, a growing number of reports describing dermatophytes can cause deep infections in diabetic and immunocompromised patients, by invading deep layers like the dermis and hypodermis. Despite the prevalence and significance of dermatophytes in clinical mycology, it is not always possible to accurately diagnose this specific infection due to its overlapping structures among species of dermatophytes. Since it is difficult to identify species that exhibit weak characteristics in the morphological highlights, identification of the dermatophyte is often relied on its morphological analysis, which is a laborious process and demands skill. The massive shift in genetic variation, the source of infection, and epidemiological research can be discovered using molecular approaches. Therefore, the development of an accurate laboratory test for dermatophyte species identification is essential for the prevention and efficient management of dermatophytoses. One such methodology allows use of PCR technology which has many methods for molecular level characterization which is rapid, efficient, and capable of producing DNA polymorphisms specific to various dermatophyte species based on distinctive band patterns seen by agarose gel electrophoresis. The RAPD-PCR approach will be used in this study protocol to molecularly characterize the dermatophytes for precise speciation of the sample. In addition to improving knowledge of fungal biology and pathology with a focus on adaptive mechanisms to combat difficult conditions from host counteractions, there is a need to improve awareness of the importance of these diseases through accurate epidemiological data. The advantages of molecular approaches for characterizing objects over traditional methods are their sensitivity and specificity.

## Introduction

Different clinical symptoms caused by dermatophytes range from superficial to subcutaneous affliction, including tinea corporis, tinea capitis, tinea pedis, and tinea unguium. ^1^ These diseases can range in severity clinically from moderate to severe depending on the host's immune system, the virulence of the strain, and other environmental factors. ^2^^–^^4^ Although dermatophytoses harm people throughout, they are more common in tropical regions due to high temperatures and humidity. ^5^ Age, sex, time of year, socioeconomic and cultural circumstances, and geographic location are all factors that might influence the development of dermatophytosis. ^6^ Potassium hydroxide (KOH) direct microscopy followed by selective medium culture is the standard procedure for dermatophyte screening in clinical samples. A quick screening technique for fungal structures is microscopy performed directly on clinical specimens, but this method lacks specificity. ^7^ By using phenotypic techniques, dermatophyte isolates can be classified according to genus or species based on colony characteristics, microscopic assessments, and biochemical tests like growth patterns. ^8^ Because there are changes from one isolate to another and overlaps in the features of several species, dermatophyte species identification by morphology in cultures may be difficult or imprecise. ^9^ Molecular-based methods rely on identifying genotypic variations in pathogenic organisms. ^10^^–^^12^ In India, a pathogen shift from *T. rubrum* to *T. mentagrophytes* has occurred alongside the appearance of novel forms of tinea that are resistant to treatment. This dermatophyte is the primary cause of tinea cruris, tinea corporis, and tinea faciei in India, exceeding prior pathogens like *T. rubrum*. ^13^^,^^14^ Molecular methods are being used to identify dermatophytes since they are more precise than conventional techniques. ^15^ The development of molecular diagnostics were encouraged by the conventional techniques for identifying dermatophytes which are imprecise and have a delayed diagnostic character. Techniques that permit for both the early and accurate detection of dermatophytosis in order to provide timely antifungal treatment that prevents generic over-the-counter medication. ^16^ Therefore, it is essential to create more reliable dermatophyte identification techniques. ^17^ The current study's objective is to utilize RAPD-PCR method to identify and distinguish between the strains of fungi present in clinical isolates that cause chronic recurrent dermatophytosis.

## Protocol

### Study design

**Oobservational study**

Study participants: 100 participants having skin lesions, hair and nail positive for dermatophytic infection will be included in the study.

### Study setting

• Samples (Skin scrapings, hair, nail clipping) will be collected from the patients visiting Dermatology OPD, Acharya Vinoba Bhave Rural Hospital, Sawangi (Meghe), Wardha.
• Samples will be processed in the Department of Microbiology, Jawaharlal Nehru Medical College, Sawangi (Meghe), Wardha.
• Method of selection of participants:
  1. Patients having skin lesions will be included for taking the skin scrapings.
  2. Patients with hair infections positive for fungal infection will be included for hair plucking.
  3. Patients with nail infection will be considered for nail clippings.
  All the samples will be collected in a black paper and stored in sterile container with proper labeling for further process.

### Eligibility criteria

Patients having visible lesions and itching, positive for fungal infections are included in the study.

### Data management

Samples will be collected directly from the patients and reports will be obtained from the microbiology laboratory. Data of all patients will be entered in Microsoft Excel taking proper precautions for wrapping the patient identifier information. The final deidentified data will be shared with statistician for further analysis.

### Sample size

Sensitivity formula for calculating the sample size.

$$N\geq Z_{\underline{1-\alpha /2}}^{\underline{2}}\frac{\text{Sens}\;\left(1-\text{Sens}\right)}{d^{2}\times \mathrm{Pr}\mathrm{ev}}$$

Alpha (α) 0.05

Estimated sensitivity (Sens) 0.95

Prevalence of disease (Prev) 0.79 ^18^

Estimated error (d) 0.05

Minimum number of diseases needed: 73

Minimum total sample size needed: 93

### Reagents required

1. Culture media Sabouraud Dextrose Agar (SDA)
2. Culture media Sabouraud Chloramphenicol + Cycloheximide Agar
3. Culture media Dermatophyte Agar Base
4. Dermato Supplement
5. Culture Media Corn meal agar
6. Distilled Water
7. Potassium Hydroxide Pellets
8. Normal Saline
9. 10% Glycerol
10. Lactophenol Cotton Blue Stain
11. Polyvinyl Alcohol
12. Ethyl Alcohol
13. Sodium Hypochlorite Solution
14. dNTP mix, 40mM
15. Hi-Temp PCR Master mix
16. Primers
17. AllPrep ^®^ Fungal DNA isolation kit
18. PCR Block Plates
19. Pipette
20. Pipette tips (10μl, 20μl, 100μl, 1000μl)
21. Microscopic glass slides
22. Coverslip
23. Inoculating loop
24. Teasing needle
25. Petri dishes
26. Forceps
27. Glass Beakers
28. Flasks
29. Glass rod
30. Bunsen Burner
31. Gel electrophoresis chamber

### Instruments required

1. Light Microscope
2. Autoclave
3. PCR
4. Biosafety Cabinet
5. Biological Oxygen Demand (BOD) Incubator
6. Electronic Weighing Machine

### Protocol

A. Sample collection
  1. The area of sample collection will be cleaned with 70% alcohol.
  2. For skin samples, the lesion will be scraped around the corners using a scalpel blade or glass slide.
  3. For nail samples, the nail clippings will be collected using a nail cutter in a sterile container
  4. For hair samples, the hair will be plucked from the shaft of the hair having a lesion.
B. Sample processing
  1. The skin scrapings will be dissolved in 10% KOH and nail clippings will be dissolved in 40% KOH for microscopic observation.
  2. Samples will be inoculated on Dermatophyte Test Medium (DTM) as well as Sabouraud's dextrose agar (SDA) containing chloramphenicol and cycloheximide.
  3. Samples will be incubated in BOD incubator, for observation of growth on the SDA and DTM slants.
  4. Lactophenol cotton blue staining and slide culture technique will be used to view morphology and colony characteristics of the samples after growth.
C. Application of RAPD-PCR method for molecular characterization
  1. Standardization of molecular assay will be done using the standard strains of Trichophyton (D15P127, CBS 118892, UCMS-IGIB-CI11), Microsporum (ATCC 36299), and laboratory-confirmed strains.
  2. AllPrep® Fungal DNA isolation kit will be used for DNA isolation from fungal cultures.
  3. The primer required for the RAPD-PCR reaction will be synthesized by Beacon designer probe/primer designer software from GeneX India Biosciences Velachery, Chennai, Tamil Nadu.
  4. PCR assay mixture, reaction buffer, dNTPs, each primer set (GACA4) and novel primer (CTGT3), DNA template, using these PCR reaction cycles will be carried out.
    (39 cycles)
    a) Denaturation at 93° - 1-minute,
    b) Annealing step at 50° -1-minute,
    c) Extension step at 72°- 1 minute
    d) Final-extension step at 72°- 7 min
  5. Final PCR products will be separated in 0.5X (Tris Borate-Ethylene diamine tetraacetic acid) Buffer and 1% Agarose and stained with the Ethidium-Bromide solution and then the image will be obtained.
  6. Interspecies and intraspecies patterns and polymorphism for known strains will be studied.

### Statistical analysis plan

All the results will be calculated using R version 4.3.2. Patients enrolled in the study sensitivity, specificity, positive predictive values (PPV), and negative predictive values (NPV) will be resulted from molecular methods used in comparison to the conventional method (gold standard).

Categorical variable will be summarized by the samples positive for fungal hyphae in KOH and culture on SDA (Sabouraud Dextrose Agar) and Dermatophyte Test Medium (DTM).

The percentage of agreement (overall, positive, and negative) between the two methods will be calculated using agreement analysis (primary and secondary endpoints), along with the Kappa coefficient, p-value, and 95% confidence interval.

**Dissemination**

Papers will be presented at relevant conferences and related studies will be published in indexed journals.

### Study status

This research is ongoing. Using previously used primers, we are validating the protocol. The instruments, reagents, enzymes, and primers are set up. Clinical sample collection and processing of the sample by conventional method and standardization of molecular methods for the identification of species is ongoing.

### Ethical considerations

This study has been granted by the ethics committee of Datta Meghe Institute of Higher Education and Research, Sawangi Meghe, Wardha with the approval number: DMIMS (DU)/IEC/2022/851, dated: 05/04/2022.

Written informed consent will be obtained from the study participants for participation in the study and publication of their data.

## Discussion

Fungal infections in humans are becoming more common, especially in immunocompromised people, which has made them a global public health concern. The course of the disease, which can range from mild cutaneous or subcutaneous infections to invasive, widespread, and potentially fatal infections, is determined by the immunological health of the host. ^19^ For epidemiological reasons, accurate antifungal treatment prevention of transmission includes exact separation between dermatophytosis and non-dermatophyte, and thorough identification of disease-causing organisms is vital. ^20^^,^^21^ The most reliable approach to diagnose dermatophytosis is through the isolation and identification of dermatophytes from clinical samples. However, it typically takes a long time for the dermatophyte to grow in culture and sporulate, which delays diagnosis. ^22^ Successful management of dermatophytes depends on prompt diagnosis and correct identification. ^23^ A study from Sweden by Ovrén, E *et al.* (2016) stated fluorescent staining method enhances the sensitivity and specificity in direct microscopy from skin, hair and nail samples and found that the specificity = (91.7–93.8%), positive predictive value (PPV) = (77.1–81.4%) and negative predictive value (NPV) = (83.7–89.9%). ^24^ Molecular methods are available for the characterization of the dermatophyte species namely restriction fragment length polymorphism (RFLP), (random amplified polymorphic DNA (RAPD), gene-specific-PCR, ^25^^,^^26^ chitin synthase encoding gene, ^27^ DNA hybridization, ^28^ and sequencing of the internal transcribed spacer region (ITS). ^7^^,^^29^ A study by Li, H. C., Bouchara *et al.* (2007) from the United Kingdom stated the use of oligonucleotide array based on ITS-1 and ITS-2 sequence for identification of 17-dermatophyte species subsequently, hybridization of a series of oligonucleotides (17–30mers) digoxigenin-labeled PCR products immobilized on a nylon membrane of the 198 clinical dermal filamentous strains tested and 90 non-targeted strains, the array sensitivity and specificity were 99.5% and 97.8%, respectively. ^30^ Many molecular methods have made a way for early detection of dermatophytes to the species level. ^31^ Along with these techniques serological methods have also detected the dermatophyte infection in the studies stated. A study by Higashi, Y *et al*. (2012) from Japan stated that the use of newly developed immunochromatographic strip-test for the diagnosis of dermatophytes found the overall sensitivity and specificity of the immunochromatographic test were 83.5% and 66.7%. ^32^^,^^33^

## Data Availability

No data are associated with this article.
