# Supplementary material for: Molecular Characterization of Dermatophyte species from rural tertiary care hospital: A Study Protocol
Source: F1000Res. 2025 Dec 31;13:136. Originally published 2024 Feb 22. [Version 4] doi: 10.12688/f1000research.141657.4 (PMC12784041; doi:10.12688/f1000research.141657.4)
Supplement: Peer review history

# Reviewer report: Reviewer response for version 4

**DOI:** 10.5256/f1000research.194482.r446383

**Published:** 2026-01-08

**Authors:** Saumya Panda

**Affiliations:** 1 Jagannath Gupta Institute of Medical Sciences and Hospital, Kolkata, West Bengal, India

The protocol in the current form may be approved.

Is the study design appropriate for the research question?

No

Is the rationale for, and objectives of, the study clearly described?

Partly

Are sufficient details of the methods provided to allow replication by others?

Yes

Are the datasets clearly presented in a useable and accessible format?

Not applicable

Reviewer Expertise:

clinical dermatology, dermatomycology

I confirm that I have read this submission and believe that I have an appropriate level of expertise to confirm that it is of an acceptable scientific standard.

# Reviewer report: Reviewer response for version 3

**DOI:** 10.5256/f1000research.193841.r443781

**Published:** 2025-12-27

**Authors:** Christophe Hennequin

**Affiliations:** 1 Sorbonne Université, Inserm, Centre de Recherche Saint-Antoine, Paris, France

Accurate identification of dermatophtosis species is an interesting topic. Currently, it is particularly important to detect Trichophyton indotineae, which frequently exhibits resistance to terbinafine. However, the rationale for this protocol and the proposed method are not suitable. For example, it states that rapid identification is desirable, but the use of a RAPD-PCR method, as proposed by the authors, will not allow for faster identification. It is also noted that the prevalence of the disease in the studied population will be 0.79, which is undoubtedly excessive. No reference method is proposed in this protocol, so the robustness of the results obtained cannot be assessed. Finally, RAPD-PCR methods are now abandoned, as many authors consider them non-reproducible. The use of MALDI-TOF mass spectrometry or targeted PCR followed by sequencing should be preferred.

Is the study design appropriate for the research question?

Partly

Is the rationale for, and objectives of, the study clearly described?

Partly

Are sufficient details of the methods provided to allow replication by others?

Partly

Are the datasets clearly presented in a useable and accessible format?

Not applicable

Reviewer Expertise:

Medical Mycology

I confirm that I have read this submission and believe that I have an appropriate level of expertise to state that I do not consider it to be of an acceptable scientific standard, for reasons outlined above.

# Reviewer report: Reviewer response for version 2

**DOI:** 10.5256/f1000research.168659.r429116

**Published:** 2025-12-11

**Authors:** Jothimani Pradeep

**Affiliations:** 1 Mahatma Gandhi Medical Advanced Research Institute, Puducherry, India

Dear Editor in Chief,

 There was no proof of that the authors have performed RAPD-PCR and other phenotypic tests to confirm the fungal isolates.

 DTM is not mentioned in the Materials & Methods.

 The data sets are not provided in the manuscript and it is hard to review this manuscript

Is the study design appropriate for the research question?

Yes

Is the rationale for, and objectives of, the study clearly described?

No

Are sufficient details of the methods provided to allow replication by others?

No

Are the datasets clearly presented in a useable and accessible format?

No

Reviewer Expertise:

Zoonotic diseases (Bacterial, fungal), sexually transmitted infections, Diabetic foot infections, Antimicrobial resistance

I confirm that I have read this submission and believe that I have an appropriate level of expertise to state that I do not consider it to be of an acceptable scientific standard, for reasons outlined above.

# Response

**Published:** 2025-12-27

**Authors:** Aditi Warghade

**Affiliations:** Department of Microbiology, Datta Meghe Institute of Higher Education and Research, Sawangi (Meghe), Wardha., Wardha, Maharashtra, India

**1. Concern: Lack of proof that RAPD-PCR and phenotypic tests were performed to confirm fungal isolates**

 We thank the reviewer for this important observation. In the revised version of the manuscript (Version 3), we have explicitly clarified that species specific primers will be designed targeting ITS region and identification will be done by Real time PCR, and phenotypic identification procedures will be  performed on all culture-positive isolates. The phenotypic confirmation will be done by macroscopic colony morphology and microscopic features on lactophenol cotton blue mount. Details regarding DNA extraction,  primer usage, amplification conditions, and gel electrophoresis have now been clearly described in the Materials and Methods section.  Representative workflow descriptions have also been added to support methodological verification.

**2. Concern: Dermatophyte Test Medium (DTM) not mentioned in Materials & Methods**

 The use of Dermatophyte Test Medium (DTM) as a screening medium for presumptive identification of dermatophytes has now been explicitly included in the Materials and Methods section, thereby ensuring completeness of the diagnostic workflow.

**3. Concern: Absence of datasets, limiting manuscript review**

 We appreciate the reviewer’s comment regarding data availability. As this manuscript is a **study protocol (we have already mentioned in the topic as well) **the complete datasets are not yet finalized. However, in response to this concern, we have now,

 -Clearly stated the data collection framework and outcome variables

 -Included a description of how datasets will be generated, stored, and analyzed

 -Added a data availability statement, indicating that datasets will be made available upon study completion. This clarification ensures integrity of an ongoing study. 

# Reviewer report: Reviewer response for version 2

**DOI:** 10.5256/f1000research.168659.r431080

**Published:** 2025-11-25

**Authors:** Dominik Łagowski

**Affiliations:** 1 Poznan University of Life Sciences, Wołyńska, Poznań, Poland

The current state of knowledge clearly indicates *that Trichophyton indotineae* is the leading cause of severe, extensive, and resistant infections in India and other regions. Why is this species not directly and explicitly discussed in the introduction as a central epidemiological problem, but only appears indirectly through citations? What is the specific contribution of the planned study to the identification and differentiation of *T. indotineae* from species of the *T. mentagrophytes/interdigitale* complex?

 Please explain how you conceive the results of the study to translate into clinical practice: how will the application of the proposed method help in identifying resistant cases (e.g., to terbinafine), modifying treatment, or monitoring the effectiveness of therapy? Do you plan to collect data on the course of treatment and clinical response to link them to the species result?

 RAPD is a technique developed many years ago, burdened with well-documented problems with repeatability and limited ability to differentiate between closely related species. Currently, the standard in dermatophyte diagnostics is to base identification on sequences (at least ITS, often also additional genes) and/or methods such as specific qPCR or MALDI-TOF MS. Why are you choosing RAPD as your primary species identification tool in 2025, rather than methods based directly on sequencing or modern molecular tests? Can you clearly compare the anticipated cost, time and technical requirements of RAPD with ITS sequencing (± other loci) in your conditions?

 To assign an isolate not only to a ‘complex’ but to a specific species, the current literature emphasises the need to analyse the ITS sequence and, often, additional markers. With this assumption, RAPD serves at best as a support technique for typing, rather than as a standalone species identification tool. How do you intend to assign isolates to specific species, especially within the *T. mentagrophytes/T. indotineae/T. interdigitale* complex based solely on RAPD patterns, without parallel sequencing?

 Anyone who has ever worked with RAPD in practice knows how difficult and time-consuming it is to establish stable PCR parameters (ionic conditions, annealing temperature, number of cycles) and electrophoresis conditions (agarose concentration, time, voltage) to obtain a reproducible and readable number of bands. Please describe how you planned the RAPD optimisation stage in detail: how many pilot rounds were conducted, how pattern stability was assessed, and what criteria must the conditions meet to be considered stable?

 Please explain how you intend to ensure the reproducibility of RAPD profiles in practice: will each sample be tested at least twice (on different days or by other operators), or do you plan to formally assess pattern consistency (e.g. similarity coefficient, cluster analysis)? How will it be documented that the banding pattern for a given isolate is stable and not a random effect of a single run?

 Comparing RAPD profiles between unknown isolates is methodologically difficult: there are no standardised reference databases, and assessing ‘similarity’ can be subjective. How do you intend to objectify the comparison of patterns: is the use of specialised software for gel image analysis and similarity coefficient calculation envisaged, or will the study be carried out exclusively ‘manually’ based on visual assessment? What similarity thresholds do you consider sufficient to conclude that two isolates belong to the same species?

 Please specify the exact composition of the panel of reference strains used for RAPD standardisation: which species does it include, how many strains per species, and does sequencing confirm any T. indotineae isolates among them? What if the RAPD profile of the tested isolate is not similar to any of the standards in the panel – how will it be classified then?

 Please clearly define what the ‘gold standard’ is in your study: is it only culture and classical morphological identification, or a combination of KOH and culture? If you consider only a situation where both KOH and culture are positive to be a ‘true case’, please explain why such a restrictive criterion was adopted and whether you are concerned about omitting clinically typical scenarios (KOH−/culture+ or KOH+/culture−).

 What specifically will you do in the event of a discrepancy between morphological identification and RAPD results (e.g., morphologically *T. rubrum*, but with a RAPD profile closer to *T. mentagrophytes*)? Is the use of a third, independent method – primarily ITS sequencing – envisaged to resolve such conflicts? If not, on what basis will you assess the accuracy of RAPD, rather than just its consistency with the morphological method?

 In the statistical section, you report the calculations of sensitivity, specificity, PPV, NPV, and the kappa coefficient. Do you plan to report 95% confidence intervals for these parameters and possibly use the McNemar test to compare pairs of methods on the same samples? How will you deal with ambiguous cases (contaminated culture, no growth, illegible RAPD profile) – will they be excluded from the analysis or assigned to specific categories, and how will this decision affect the sensitivity/specificity estimates?

 Have you considered referring to existing standards and recommendations in the text (e.g. CLSI documents, ESCMID/ECMM recommendations, ISHAM comments on *T. indotineae* and the need for sequencing methods)? How does the proposed approach fit in with these recommendations, particularly about the identification of problematic species and the surveillance of drug resistance?

 Since the starting point is the problem of chronic, resistant infections, why does the protocol not provide for any form of drug susceptibility testing (MIC tests, even for selected isolates) or at least systematic archiving of isolates for later analysis of resistance genes? Does this not significantly limit the practical value of species identification, since it is not accompanied by information on potential resistance?

 Please specify exactly what quality control elements will be used in the molecular part: will each RAPD series include a positive control, a negative control and an extraction control; will each sample be analysed in duplicate or triplicate; how will you document the absence of contamination and the stability of the profiles?

Is the study design appropriate for the research question?

No

Is the rationale for, and objectives of, the study clearly described?

Partly

Are sufficient details of the methods provided to allow replication by others?

No

Are the datasets clearly presented in a useable and accessible format?

Partly

Reviewer Expertise:

mycology, veterinary mycology, micorbiology, resistance

I confirm that I have read this submission and believe that I have an appropriate level of expertise to state that I do not consider it to be of an acceptable scientific standard, for reasons outlined above.

# Reviewer report: Reviewer response for version 2

**DOI:** 10.5256/f1000research.168659.r300374

**Published:** 2024-07-22

**Authors:** Saumya Panda

**Affiliations:** 1 Jagannath Gupta Institute of Medical Sciences and Hospital, Kolkata, West Bengal, India

I thank the authors for their response. My comments on the current version are as follows:

 1. While I appreciate the deletion of the characterization of  dermatophytosis in the study as 'chronic and recurring', the subtitle "an observational study" is neither here nor there. While the earlier description of the study design as 'case-control' was plainly incorrect, the correct description would have been "a cross-sectional study". It is to be noted that 'case control' as well as 'cross-sectional' studies are all observational ones.

 2. The reference given for the calculation of sample size is  of a review article published in 2008. Other than being outdated, it is not possible to decipher exactly which Indian study has been taken as the prevalence reference.

 3. The rationale of the study still remains inadequately addressed. One of the two references cited as explanation of the current epidemic was published in 1995!!!!

Is the study design appropriate for the research question?

No

Is the rationale for, and objectives of, the study clearly described?

Partly

Are sufficient details of the methods provided to allow replication by others?

Yes

Are the datasets clearly presented in a useable and accessible format?

Not applicable

Reviewer Expertise:

Dermatology

I confirm that I have read this submission and believe that I have an appropriate level of expertise to state that I do not consider it to be of an acceptable scientific standard, for reasons outlined above.

# Reviewer report: Reviewer response for version 2

**DOI:** 10.5256/f1000research.168659.r300373

**Published:** 2024-07-16

**Authors:** Hassan Aboul-Ella

**Affiliations:** 1 Cairo University, Giza, Giza Governorate, Egypt

The respected authors responded clearly to the previously addressed comments and I don't have any further comments at this stage.

Is the study design appropriate for the research question?

Yes

Is the rationale for, and objectives of, the study clearly described?

Yes

Are sufficient details of the methods provided to allow replication by others?

Yes

Are the datasets clearly presented in a useable and accessible format?

Not applicable

Reviewer Expertise:

Mycology, dermatophytosis, and mycological diagnostic techniques are my major research areas

I confirm that I have read this submission and believe that I have an appropriate level of expertise to confirm that it is of an acceptable scientific standard.

# Reviewer report: Reviewer response for version 1

**DOI:** 10.5256/f1000research.155121.r256151

**Published:** 2024-05-14

**Authors:** Saumya Panda

**Affiliations:** 1 Jagannath Gupta Institute of Medical Sciences and Hospital, Kolkata, West Bengal, India

This is an interesting and relevant protocol, given that there is an epidemic of chronic and recurrent dermatophytosis that is currently ongoing in our country as well as in several other countries and regions in the world. However, the protocol suffers from some serious lapses:

 1. The protocol is titled as 'Study of molecular characterization for diagnosis of chronic and recurrent dermatophytosis'. However, the sampling methods and selection criteria of the cases do not mention these conditions at all!

 2. Study design is mentioned as 'case control'. However, there is no description of the control group. The description matches that of a cross-sectional study, which is what it should be.

 3. In sample size estimation, there is no reference to the prevalence value that has been taken, thus making the whole exercise infructuous.

 4. The basic rationale of the study has not been mentioned - the current epidemic of recalcitrant dermatophytosis is due to the emergence of a new species called *Trichophyton indotineae*, which is morphologically and culturally indistinguishable from *Trichohyton mentagrophytes* and *Trichophyton interdigitale.* Thus its identification requires advanced molecular diagnostics like ITS genotyping or modified MALDI-TOF, which are not frequently available.

Is the study design appropriate for the research question?

No

Is the rationale for, and objectives of, the study clearly described?

Partly

Are sufficient details of the methods provided to allow replication by others?

Yes

Are the datasets clearly presented in a useable and accessible format?

Not applicable

Reviewer Expertise:

Dermatology

I confirm that I have read this submission and believe that I have an appropriate level of expertise to confirm that it is of an acceptable scientific standard, however I have significant reservations, as outlined above.

# Response

**Published:** 2024-06-29

**Authors:** Aditi Warghade

**Affiliations:** Department of Microbiology, Datta Meghe Institute of Higher Education and Research, Sawangi (Meghe), Wardha., Wardha, Maharashtra, India

Respected Madam,

 Thank you for considering our manuscript and suggesting the following changes to be processed for further publication in F1000 research subject to a satisfactory revision as per your suggestions. We have made the changes in our manuscript. Please consider the following changes.

 This is an interesting and relevant protocol, given that there is an epidemic of chronic and recurrent dermatophytosis that is currently ongoing in our country as well as in several other countries and regions in the world. However, the protocol suffers from some serious lapses:

 1. The protocol is titled as 'Study of molecular characterization for diagnosis of chronic and recurrent dermatophytosis'. However, the sampling methods and selection criteria of the cases do not mention these conditions at all!

**Answer**: Thank you for the suggestion mam. We have changed our title to "Diagnosis and Molecular Characterization of Dermatophytosis: An Observational Study". Track changes enabled and highlighted for the same.

 2. Study design is mentioned as 'case-control'. However, there is no description of the control group. The description matches that of a cross-sectional study, which is what it should be.

**Answer**: Thank you for the suggestion, mam. We have stated the study design as an observational study as suggested.

 3. In sample size estimation, there is no reference to the prevalence value taken, thus making the whole exercise infructuous.

**Answer**: Thank you for the suggestion mam. Mistakenly the study wasn’t added in the prevalence section. I have corrected the draft and added the study with Ref no-3 and highlighted the citation as it is not getting added in the reference list in sequence.

 4. The basic rationale of the study has not been mentioned - the current epidemic of recalcitrant dermatophytosis is due to the emergence of a new species called *Trichophyton indotineae*, which is morphologically and culturally indistinguishable from *Trichophyton mentagrophytes* and *Trichophyton interdigitale.* Thus its identification requires advanced molecular diagnostics like ITS genotyping or modified MALDI-TOF, which are not frequently available.

**Answer**: Thank you for the suggestion, mam. I have added the reference information in the draft and quoted it as Ref no 1&2.

# Reviewer report: Reviewer response for version 1

**DOI:** 10.5256/f1000research.155121.r251787

**Published:** 2024-03-27

**Authors:** Hassan Aboul-Ella

**Affiliations:** 1 Cairo University, Giza, Giza Governorate, Egypt

The current work describes a protocol for an observational study through collecting samples from dermatophytes-suffering patients followed by conventional and molecular diagnostic process, the obtained data will be very useful in improving and updating the epidemiological data in this field.    

**General comments**

 While the research focuses on an encouraging area of research and addresses an area of study with minimal existing research, it requires substantial editing of vocabulary in English especially in the following sections; abstract, introduction, and discussion.

**Specific comments**

 - The title needs certain modifications to be more representable for the designed study. Therefore, I suggest "Diagnosis and molecular characterization of dermatophytosis: an observational study"

 - The abstract, introduction, and methodological protocol sections are well designed, structured, and described.  

 - The discussion is missing certain necessary references to support the flow of the work and the researcher point of view in the following points;

**1)** The most reliable approach to diagnose dermatophytosis is through the isolation and identification of dermatophytes from clinical samples. However, it typically takes a long time for the dermatophyte to grow in culture and sporulate, which delays diagnosis. 

 - The suggested [Ref 2] ****in which a culture-dependent cross sectional study has been performed on dermatophytosis showing the importance of culture in dermatophytosis diagnosis as well as the extend of to which extend this technique is laborious and time consuming and that will support the researcher point of view. 

**2)**  Many molecular methods have made a way for early detection of dermatophytes to the species level.

 - The suggested [Ref 3] ****in which a comprehensive and collective mentioning and illustration of the molecular advancement and molecular diagnostic techniques that is have been developed in the field of dermatophytosis and the extend of the diagnostic level of those techniques and that of course will strongly support the researcher point of view. 

**3) **A study by Higashi, Y *et al*. (2012) from Japan stated that the use of newly developed immunochromatographic strip-test for the diagnosis of dermatophytes found the overall sensitivity and specificity of the immunochromatographic test were 83.5% and 66.7%. 

 - The suggested [Ref 1] with all respect of the cited study as Higashi *et al. *2012, study in the field of immunochromatographic diagnosis of dermatophytosis is really a pioneering study in this field but to be more accurate and more updated I suggest adding the suggested reference in addition to the Higashi *et al. *2012, study as the suggested reference represents a newest designed and developed immunochromatographic assay for dermatophytosis diagnosis with a competitive results and certain important production modification over that of Higashi kits.

Is the study design appropriate for the research question?

Yes

Is the rationale for, and objectives of, the study clearly described?

Yes

Are sufficient details of the methods provided to allow replication by others?

Yes

Are the datasets clearly presented in a useable and accessible format?

Not applicable

Reviewer Expertise:

Mycology, dermatophytosis, and mycological diagnostic techniques are my major research areas

I confirm that I have read this submission and believe that I have an appropriate level of expertise to confirm that it is of an acceptable scientific standard, however I have significant reservations, as outlined above.

## References

[1] : Development, preparation, and evaluation of a novel dotted lateral flow immunochromatographic kit for rapid diagnosis of dermatophytosis. *Sci Rep* .2023;13(1) : 10.1038/s41598-023-27443-4 248 10.1038/s41598-023-27443-4 36604481 PMC9816107

[2] : Cross-Sectional Study on Dermatological Affections of Companion Animals Caused by Dermatophytes and other Keratinophilic Fungi in Greater Cairo Area, Egypt. *Advances in Animal and Veterinary Sciences* .2020;9(4) : 10.17582/journal.aavs/2021/9.4.615.622 10.17582/journal.aavs/2021/9.4.615.622

[3] : Recent trends in rapid diagnostic techniques for dermatophytosis. *Int J Vet Sci Med* .2020;8(1) : 10.1080/23144599.2020.1850204 115-123 10.1080/23144599.2020.1850204 33426048 PMC7751388

# Response

**Published:** 2024-06-29

**Authors:** Aditi Warghade

**Affiliations:** Department of Microbiology, Datta Meghe Institute of Higher Education and Research, Sawangi (Meghe), Wardha., Wardha, Maharashtra, India

Respected sir,

 Thank you for considering our manuscript and suggesting the following changes to be processed for further publication in F1000 research subject to a satisfactory revision as per your suggestions. We have made the changes in our manuscript as per the suggestions given. Please consider the following changes

 The title needs certain modifications to be more representable for the designed study. Therefore, I suggest "Diagnosis and molecular characterization of dermatophytosis: an observational study"

**Answer:**

 Thank you for the suggestion, sir. I agree with the changes suggested and would like to change the title to "Diagnosis and Molecular Characterization of Dermatophytosis: An Observational Study"

 - The abstract, introduction, and methodological protocol sections are well-designed, structured, and described.  

 - The discussion is missing certain necessary references to support the flow of the work and the researcher's point of view in the following points;

**1)** The most reliable approach to diagnose dermatophytosis is through the isolation and identification of dermatophytes from clinical samples. However, it typically takes a long time for the dermatophyte to grow in culture and sporulate, which delays diagnosis. 

 - The suggested [Ref 2] ****in which a culture-dependent cross-sectional study has been performed on dermatophytosis showing the importance of culture in dermatophytosis diagnosis as well as the extend of to which extend this technique is laborious and time consuming and that will support the researcher point of view. 

**Answer:** Thank you for the comment, sir. We have added the suggested reference in the draft, quoted as Ref No-4, and highlighted the citation as it is not getting added in the reference list in sequence.

**2)**  Many molecular methods have made a way for early detection of dermatophytes to the species level.

 - The suggested [Ref 3] ****in which a comprehensive and collective mentioning and illustration of the molecular advancement and molecular diagnostic techniques that is have been developed in the field of dermatophytosis and the extend of the diagnostic level of those techniques and that of course will strongly support the researcher point of view. 

**Answer:** Thank you for the suggestion, sir. We have added the suggested reference in the draft quoted as Ref No- 5, and highlighted the citation as it is not getting added in the reference list in sequence.

**3) **A study by Higashi, Y *et al*. (2012) from Japan stated that the use of newly developed immunochromatographic strip-test for the diagnosis of dermatophytes found the overall sensitivity and specificity of the immunochromatographic test were 83.5% and 66.7%. 

 - The suggested [Ref 1] with all respect of the cited study as Higashi *et al. *2012, study in the field of immunochromatographic diagnosis of dermatophytosis is really a pioneering study in this field but to be more accurate and more updated I suggest adding the suggested reference in addition to the Higashi *et al. *2012, study as the suggested reference represents a newest designed and developed immunochromatographic assay for dermatophytosis diagnosis with a competitive results and certain important production modification over that of Higashi kits..

**Answer**: Thank you for the suggestion, sir. We have added the suggested reference in our study and quoted it as Ref No-6, and highlighted the citation as it is not getting added in the reference list in sequence.
